# Links between types of value orientations and consumer behaviours. An empirical study

**DOI:** 10.1371/journal.pone.0264185

**Published:** 2022-02-24

**Authors:** Grzegorz Adamczyk, Jorge Capetillo-Ponce, Dominik Szczygielski

**Affiliations:** 1 Institute of Sociology, The John Paul II Catholic University of Lublin, Lublin, Poland; 2 Institute of Sociology, University of Massachusetts at Boston, Boston, Massachusetts, United States of America; Aalborg University, DENMARK

## Abstract

The present study concerns the phenomenon of the co-dependence of value orientations and consumer behaviours. Although the idea of the overall connections between both areas is not new, the article contributes to the knowledge about relations between very specific consumer behaviours and social value orientations among people married or in an informal partnership. Using the obtained data, we examine to what extent the prosocial, reciprocal, and egoistic value orientations coexist with compensative, compulsive, and demonstrative buying. The analysis confirms the hypotheses that representatives of the reciprocal and egoistic orientations show susceptibility to compensative and compulsive buying to a greater extent than persons preferring other value orientations. In addition, the data show co-dependence of the reciprocal value orientation and demonstrative buying, while the prosocial value orientation seems to protect against compulsive buying. According to the regression analysis, this effect disappears if prosocially oriented persons suffer from low self-esteem at the same time being characterised by strong materialism. All hypothesised relations between value orientations and consumer behaviours have been analysed in multidimensional models considering materialism, self-esteem, age, and gender as the main factors of compensative and compulsive buying. The findings come from the 2017 survey based on a statistically representative sample of 1,121 adult Poles who were then married or in an informal partnership. The German Compulsive Buying Indicator and Lange’s Scale of Demonstrative Buying were introduced to measure consumer behaviours.

## Introduction

Sociologists have discussed the links between value orientations and consumer behaviours for a long time. Already Weber [1] pointed to the relation between the Protestants’ specific ethos and consumer behaviours within the capitalist economy. Both phenomena found a common denominator–vocational work coupled with professional development and extension of economic capital as evidence of God’s favour should guide towards the transcendent goal, salvation. Hence, the Protestant ethic appreciated some values such as diligence, entrepreneurship, discipline, thriftiness and it legitimized the struggle for wealth using the parallelly developed capitalist rationality of economic activities. This synergy of religiously motivated value orientations and the capitalist economy led to a significant economic growth in Protestant countries [2].

In a certain sense, the above outlined relation is reflected in the contemporary consumer society. Although the transcendental goal of mundane life in this type of society disappeared, the social role of consumer behaviours is still the same. The Protestants described by Weber used consumer goods as symbols of God’s favour, which guaranteed them the social prestige. Contemporary consumers use goods as socially desired symbols of meaning, satisfaction and self-actualization in consumer society [3] which promotes the consumerist lifestyle or even pressurises into practising it [4].

Independently of the overall relations between values and consumption integrated e.g. in practised lifestyles [5, 6], researchers find different links between value orientations and particular consumer behaviours. E.g. Marcén and Morales [7] found out that culture understood as a set of different values, beliefs, traditions etc. is a significant factor in home-ownership. The probability of being a homeowner is greater among those US immigrants whose counterparts in the countries of origin prefer home-ownership. Ihsan, Seonggoo and Chankoo [8] showed that the social importance of health values corresponds to more positive buying attitudes towards green products. Lange [9] pointed out a higher level of debts among the 15–25 year-olds rejecting the self-actualization values (e.g. creativity, spontaneity, self-containment, participation) than among the youth preferring these values.

Based on the literature review, we believe that overall dispositions to assess and to act in interdependent situations (social value orientations) are related to specific types of consumer behaviours. In this paper, we focus on compulsive, compensative, and demonstrative buying. So far, this relation has not been examined within empirical projects conducted on countrywide samples. Hence, this study will contribute to the existing literature on the new aspect of the relationship between social value orientations and consumer behaviours.

A broader discussion about disorders connected with buying began at the end of the 1980s triggered by the study conducted by Faber, O’Guinn and Krych [10]. Since then, studies on this kind of disorders have been conducted on two levels: those referring to consumer research and those based on clinical psychology and psychiatry. The latter treat the disorders named e.g. compulsive or pathological buying as a behavioural addiction, while consumer research describes the disorders as an irrational way of purchasing [11]. The last-mentioned approach is a reference point for our study. The results of the initial analyses presented elsewhere [12] showed that susceptibility to compulsive buying accompanies increasing materialism, worsening self-esteem, and lowering age. What is more, women display a stronger predisposition to compulsive buying than men. The results of the regression analysis show that a high level of materialism and low self-esteem are the most important factors of compulsive buying, while the role of age or gender is relatively marginal. Most studies on compulsive buying including our initial analyses from 2020 are focused rather on recognition of psychical or sociodemographic factors of compulsive buying while value orientations remain rather ignored. Now we would like to bridge the gap and prove that susceptibility to compulsive buying is explained by value orientations. Because compensative and demonstrative buying co-exists with compulsive buying, the analyses will be additionally extended with these types of consumer behaviours.

We expect relatively strong bonds between value orientations and compulsive, compensative, and demonstrative buying for several reasons. One of them is sociologists’ observation concerning the development of consumer society promoting consumerist lifestyle and life strategies [4]. During the process, values become more and more inherent in consumer goods due to the process of branding, which is an important element of marketing strategies. Values are decision criteria legitimized by an individual more or less consciously and applied more or less true to social expectation [13]. On the one hand, these criteria are an important factor shaping consumer behaviours due to consumer norms transmitted in the socialization process. On the other hand, cultural values contained in material goods perform five basic functions influencing consumers’ behaviours. Consumers make use of the symbolic value of goods to demonstrate the real or expected social status, to confirm their own social competence, to manifest their identity, to compensate for the problems of everyday life, or to create pleasure experiences [14]. Material goods containing cultural values perform an especially important role in consumer society, because its members believe that “meaning and satisfaction in life are to be found through the purchase and use of consumer goods” [3, p. 4].

To what extent consumers take advantage of the above functions of cultural values inherent in material goods depends more or less on value orientations. The connections between value orientations and consumer behaviours are unavoidable because people’s judgments in one area are more or less coupled to judgments in other areas, and value orientations as a specific socio-cultural “baggage” characterising the social environment, social group or an entire society can spill over into other areas of life including consumer behaviours [15].

The remaining part of this paper is organised as follows: at the beginning, we deal with the theoretical framework and define the types of value orientations and consumer behaviours which we are interested in. Then, we describe what we expect putting four hypotheses and seeking justification for them. In the next section, we present the methodological basics of our empirical study and the data used to verify the hypotheses. Finally, we conclude in a critical discussion about the potential reasons for the observed relations between value orientations and consumer behaviours. Remarks about the limitations of the study end our paper.

## Conceptual framework

### Types of value orientations

The concept of social value orientations explains people’s disposition to assess and to act in interdependent situations. This disposition differs individually depending on a person’s preference concerning what kind of relation between their own and other people’s good they are ready to accept. These individual preferences can be assigned to more or less coherent types placed on the axis of two extremes: prosocial and individualistic value orientations [16]. Prosocials try to maximize the outcomes for themselves and others and/ or to minimize the differences between the outcomes. The prosocial value orientation is based on the principle of other people’s good, including the norms which encourage pure altruistic behaviours or giving up one’s own good for the benefit of other people in need. Or more generally, prosocials are able to give up their personal interest if a conflict occurs between their own interest and other people’s interest. The well-being of others in need is a central value of prosocials [17, 18]. This prosocial susceptibility is based on the norm of social responsibility which makes an individual help other persons who require aid. People’s susceptibility to aid activities increases along with the growing consciousness that somebody’s well-being is dependent on their help [19]. The probability of aiding activities is greater when an overall social norm becomes a personal norm understood as a personal duty to act in a specific way in a given situation. In other words, a personal norm is a specific operationalization of a social norm which shows in which way the social norm should be realised. An individual fulfilling the personal norm of aid directed to the needy people can experience satisfaction with themselves [20]. Pletzer at al. [16] mention a considerable number of studies which characterise prosocials as persons donating more and showing civic attitudes as well as engagement in volunteering and in pro-environmental activities to a greater extent than other people.

Persons oriented individualistically prefer the maximization of their own outcomes with little or no respect for others’ outcomes. Individualists are characterised by focus on their own individual sphere, putting their own good above other people’s good, indifference towards the interests of other people, and insensitivity to social issues. In the literature, individualists are often combined with the segment of so-called competitors in a common type of proselfs. Mainly, competitors are interested in maximization of the relative difference between their own outcome and the outcome of others [16, 17]. Because proselfs consciously give up any contribution to other people’s good, even if they have such a possibility, this disposition to assess and to act in interdependent situations can be defined as egoistic orientation contrary to prosociality.

According to Peter Blau’s social exchange theory [21], individuals perform social acts to reciprocate somebody’s previous act in the belief that they contracted a kind of debt with the person whose acting generated an economic, social or psychical profit for them. As usual, the person in debt is socially obligated to repay the act someday proportionally to the previous profits. This exchange of profits might be direct such as an exchange of neighbourly help, or indirect e.g. if a donation is not exchanged for poor people’s gratitude but for social recognition among representatives of a higher social stratum. Although the mechanism of social exchange is common, its scheme is different depending on the social value orientation.

Based on the social exchange theory, prosocials might be split into two segments: prosocials in the narrow sense and reciprocals (We use this word to refer to people who are inclined to reciprocal behaviours). Representatives of the first group act really altruistically accepting potential inequality of profits in somebody’s favour. Reciprocals try to minimize the differences of the exchange partners’ outcomes caring for the balance between their own good and other people’s good. On the one hand, they are capable of acting prosocially but only being convinced that the exchange partner will be able to return the favour in the future and “the value” of the repayment will be the same. On the other hand, reciprocals are able to accept a prosocial act directed to them if they positively assess their future ability to repay the act by doing something of comparable importance for the exchange partner. The orientation reflects a specific defence mechanism which makes people maintain good relationships just in case of crisis situations and the necessity of receiving other people’s help. The main motif of reciprocal social actions including help directed to people in need is the expectation that the actions will be repaid in the future, although the reasons for the repayment can be quite different [18].

The reciprocal exchange resembles the most an economic exchange when partners know exactly the potential profits beforehand if the exchange should come through (e.g. a purchase of a car). Similarly, reciprocals expect that a favour should be returned by an exchange partner at the first opportunity and the returned favour should be of the same weight. For this reason, reciprocals prefer social exchange conducted directly rather than indirectly. This rule is ignored by prosocials. Although they take part in the social exchange game, they expect neither a direct repayment of their prosocial acts nor a repayment valued on the same level. In addition, they count on the possibility that their prosocial act will not be repaid at all directly or even they want to avoid a direct repayment.

In what way might the nature of the egoistic value orientation be explained compared with the prosocial and reciprocal variants? At first sight, the egoists’ social exchange seems to be a reverse of the prosocials’ approach. But this statement needs an additional explanation. Egoists shape the social exchange in such a way that their profit which ends the exchange process should be greater than the profit of the exchange partner. They achieve this goal in two ways. Firstly, they break the basic reciprocal principle: the receiver of an act bringing them profits should repay it on the same level in the near or further future. Secondly, even though egoists initiate the social exchange, they do it to provoke a reciprocal act of their exchange partners which finally is not repaid or repaid but on an insufficient level. The social exchange preferred by egoists resembles economic activities on the free market based on the rational choice principle maximizing the profits and minimizing the incurred costs at the same time [22].

### Types of consumer behaviours

According to Antonides and van Raaij [23], the term of consumer behaviour is very capacious and embraces mental and physical acts including their meanings, motives, and causes. These acts serve the achievement of consumer goals, which has different individual and societal consequences. The double meaning of the term is especially worth underlining. On the one hand, a consumer behaviour is a direct observable action like buying something. On the other hand, a consumer behaviour is a set of different meanings, senses, and motivations reflecting cultural values which are hidden in consumer goods and which actually guide the display of consumer behaviours.

Consumer behaviours compose the domains which are “sets of behaviours frequently organized around a common goal” [23, p. 178]. These sets include cultural values of consumer goods which create connections between goals of consumer behaviours and concrete acts facilitating the achievement of the goals. For this reason, different types of consumer behaviours should correspond to different types of goals rooted in value orientations. For example, the goal of compensation may be realised by means of compensative buying. The goal of consumer confirmation and hedonistic experience may be achieved on the basis of the domain of compulsive buying. Other goals such as social positioning and expression may be reached due to demonstrative buying. Because the goals of consumer behaviours can be linked with value orientations, the question about empirical relationships between the types of social value orientations and consumer behaviours seems to be justified.

Although the phenomenon of compulsive buying was noticed by Kraepelin [24] at the beginning of 20th century, it was only the study by Faber, O’Guinn, Krych from 1987 that began a broader discussion about the nature and determinants of the phenomenon’s presence. Definitions of compulsive buying show two basic traits. Firstly, compulsive buying serves as an escape from the reality of everyday life; and secondly, the person who buys compulsively is made to do this because of the experienced psychical pressure. The consciousness of the negative consequences is not sufficient protection against actions of this type [25]. O’Guinn and Faber define compulsive buying as “chronic, repetitive purchasing that occurs as a response to negative events or feelings” [26, p. 149]. The act of purchase delivers pleasure and relief, but then they are replaced by the feeling of guilt and remorse when the consciousness of irrationality of the act appears [11].

Compulsive buying can be understood as a behavioural addiction in three basic ways: as an impulse control disorder, an obsessive-compulsive disorder or a neuroadaptive process. In the first case, the compulsive buyer loses control over impulses on account of an increasing tension or anxiety which can be replaced by the experience of pleasure, satisfaction or relax only due to buying. Treating compulsive buying as an obsessive-compulsive disorder means that a compulsive buyer experiences an internal pressure to buy, which reduces unpleasant psychical states. From the neuroadaptive point of view, compulsive buying is experienced by the brain as a reward. An excessive repetition of the rewarding purchase leads to the formation of the neuroadaptive mechanism analogous to the development of substance abuse [27]. The undertow of compulsive buying can be an individual’s difficulties in defining themselves, which brings the consideration of different substitutes for identity deficiencies [28]. Although compulsive buying is experienced momentarily as a real compensation for the problems in different life areas, the long-term consequences of the behaviour are negative psychologically (guilty feelings, weak self-esteem), socially (disordered interpersonal relationships, conflicts), and economically (debts) [26].

From the point of view of the Rational Choice Theory, compulsive buying can be found to be a type of an irrational consumer behaviour as opposed to compensative, demonstrative, and rational buying. Although the post-modern paradigm inclines towards the conclusion that each consumer constructs their own rationality, the application of the principle of maximizing the expected utility might be helpful to fix the clear borderlines between compulsive buying on the one hand and compensative, demonstrative, and rational buying, on the other. In general, consumers choose this behaviour from a set of the accessible options, which maximizes the profits or minimalizes the incurred costs [22]. Hence, compulsive buying is irrational because costs of the behaviour are higher than profits. Although a compulsive buyer experiences a desirable relief of tension and an improved frame of mind resulting in higher self-esteem (profits), the potential negative consequences (costs) are significantly more complex–loss of self-control to a greater or lesser extent, psychical suffering from the returning remorse and feelings of guilt, withdrawal symptoms, excessive expenditures and debts of the household leading to private bankruptcy, crisis of interpersonal relationships, interrupted professional career, comorbidity of behavioural or substance addiction or even problems with the law [29].

Although the borderline between compulsive and compensative buying is fixed by the balance between the profits and the costs, sometimes both buying styles overlap. Assuming that compulsive buying is a kind of behavioural addiction, the compensation experienced during purchase constitutes the object of the addiction. In this sense, compulsive buyers always purchase compensatively at the same time. In contrast, compensative buying might be an autonomous purchase style which may precede the stage of compulsive buying but it does not have to lead to it [30]. This idea is the basis of the Compulsive Buying Scale distinguishing three types of buyers placed on the continuum: rational–compensative–compulsive buyers [26]. Compensative, like compulsive buying does not primarily aim to make use of the functional utility advantages of a given item but to compensate for the problems occurring in quite different life areas. Compensative buyers experience satisfaction based on a purchasing act, which is able to reduce internal tension and strengthen their self-esteem [31]. However, the costs of the compensation never exceed the profits.

The roots of demonstrative buying are in the need of social prestige. Demonstrative buyers, like compensative buyers purchase the given items to use their attributes to achieve goals quite different from the utility functions of the items. In case of demonstrative buying, improvement of one’s own social status and self-esteem is the primary benefit. Hence, demonstrative buyers buy those goods which are perceived socially as fashionable, exclusive and desired. For this reason, brands play a special role for them, which in fact is taken advantage of by marketers very willingly. Like in case of compensative buying, the desire for demonstrative purchase can turn into compulsive buying if the balance between costs and profits is upset and the psychical, social, and economic costs exceed the profits in the form of an improved social status.

## Research aim

The goal of the study was an attempt to answer the question if correlations between the above defined types of value orientations and consumer behaviours exist. The following hypotheses will be tested:

H1: The stronger the prosocial value orientation, the stronger susceptibility to compensative buying
H2: The stronger the reciprocal value orientation, the stronger susceptibility to compensative/ compulsive buying
H3: The stronger the egoistic value orientation, the stronger susceptibility to compensative/ compulsive buying
H4: The stronger the reciprocal value orientation, the stronger susceptibility to demonstrative buying.

A further analysis intends to verify whether the correlations between value orientations and consumer behaviours appear or disappear if the variables describing the main factors of compulsive buying such as materialism, self-esteem, age and gender are included into the multidimensional model.

The assumed hypotheses have their roots in the theoretical concept of the social value orientation and social exchange. Prosocials take into consideration the interest of other people if a conflict occurs between their own interest and other people’s interest. Prosocials are altruistically oriented, which means their acceptance of inequality in the process of the social exchange. Some researchers indicate that purely altruistic motifs of prosocial behaviour do not exist. More or less, egoistic components are hidden in altruistic behaviours; it might be an appreciation of the social environment or an increasing level of the brain neurotransmitters responsible for the pleasure feelings. The deficiency of the expected co-results of the altruistic behaviours might lead to decreasing self-esteem, sometimes resulting in different behavioural addictions [32, 33]. Certainly, compensative buying belongs to the catalogue of possible responses to the insufficient co-effects of altruistic acts.

As mentioned, reciprocals expect that their prosocial activity will be repaid by the exchange partner on the same level in the near or further future. The better social position of reciprocals is, the bigger future reciprocated benefits might be expected. Repaid benefits should be proportional not only to the primary prosocial act, but to the social position of reciprocals. For this reason, reciprocals can try to improve their social position to have more benefits in the future if they decide to act prosocially. In consumer society, a demonstrative consumer behaviour is a relatively easy way to improve the social position thanks to the purchase of goods perceived as socially desired such as brands. People reciprocating the received profits have hopes for the future winnings in the reciprocal cycle. In more complex social structures, social norms replace the direct exchange between individuals. Then, the conformity referring to the social expectations included in the social norms provide social acclaim for an individual. Because consumer society is permeated with consumerism, demonstrative buying and consumption are the best way to gain the social prestige true to the social expectations. Although demonstrative buying and consumption do not lead to a direct social exchange with other members of consumer society, reciprocals gain good reputation which might be profitable in the future social exchange. Like the wealthy persons who donate for charity to gain social reputation among the representatives of their social class and not to win profits from poor people directly, reciprocals buy demonstratively to strengthen their social position, which can be paid off in the future [21].

We expect a co-dependence of the reciprocal value orientation and demonstrative buying as well as compensative/ compulsive buying. On the one hand, a reciprocal act of a debtor might not occur. If a person expecting the act is oriented reciprocally, they might seek compensation rooted in compensative buying. On the other hand, especially in a consumer society, demonstrative buying, aiming to improve the social position for future reciprocal acts, can become compulsive. Over-average usage of the symbolic values of consumer goods to gain social prestige and to improve social positioning can lead to susceptibility to demonstrative buying which is difficult to hold back. If the costs of the acts began to exceed the profits, it means that demonstrative buying turns into compulsive buying.

Egoists represent quite a different social orientation than prosocials. As mentioned, egoists are characterised by focus on their own interest without consideration of other people’s interest [34]. In addition, competition with other individuals is a crucial element of the orientation; egoists pursue the aim continuously to increase the differences between their own achievements and the outcomes of other people. Hence, cooperation with others is a sign of weakness for egoists; only competition is a sign of real power for them [35]. But this strategy can bring specific consequences, especially in consumer society where competition consists in an acquisition of consumer symbols indicating a high social position and an advanced extent of consumeristic self-realization. Competition in this area leads to the belief that “meaning and satisfaction in life are to be found through the purchase and use of consumer goods” [3, p. 4]. Permanent success in the area is not possible to maintain. A consumer is threatened by the danger of somebody turning out better in the competition and achieving a higher level of prestige and self-realization. Compensative buying can be an easily accessible antidote for the situation. Shopping destinations are places where consumers compete especially strongly. These are the places where consumers meet and can demonstrate their status based on purchase acts. Consumer goods play a less important role–brand of the store, prestige of the shopping destination, luxurious design of the store, special treatment of clients by the staff are the essence. It is an ideal place for egoists who need to compete with other consumers constantly. This coupling of these consumers’ specific needs with the shopping destination is an ideal basis for the growth of susceptibility to compulsive buying. According to researchers dealing with the phenomenon, the subject of compulsive buying is not a consumer good, but experience connected with a purchase act [36]. Hence, we assume a positive correlation between the egoistic value orientation and compulsive buying.

## Materials and methods

### Sample

The survey about compulsive buying was conducted in Poland in 2017 on the statistically representative sample of 1,121 adult Poles married or in an informal partnership (Table 1). Once the survey had been approved by the Ethical Committee of the Institute of Sociology at the Catholic University of Lublin (decision 2/2017), the study was carried out using Computer Assisted Web Interview at the respondent’s home. The fieldwork was conducted by the Institute of Market and Public Opinion Research GfK Polonia which was responsible for the recruitment process of participants coming from the GfK internet panellists. The consent of participants were ticked by them in the questionnaire. The decision about the sample size of the survey based on the assumption that the statistical error should not exceed the level of 3% which is commonly accepted in the social research. The 2017 adult population of Poland amounted to 31.5 million of people, 64.1% of them were married or in an informal partnership (20.2 million). Taking into consideration the size of the target group, the sample of about 1,110 respondents meets the condition of the statistical error on the maximal level of 3%.

**Table 1:** Sociodemographic characteristic of the sample.

	*N =*	%
**Gender**		
**Male**	*557*	49.7
**Female**	*564*	50.3
**Age**		
**18–29**	*129*	11.5
**30–39**	*274*	24.4
**40–49**	*254*	22.6
**50–59**	*223*	19.9
**60–64**	*108*	9.6
**65 +**	*133*	11.9
**Average age**	46.6	
**Family status**		
**Formal marriage**	*923*	82.2
**Informal partnership**	*199*	17.7
**Children**		
**The respondent does not have children**	*176*	15.7
**One child**	*361*	32.2
**Two children**	*453*	40.4
**Three children or more**	*131*	11.7
**Professional status**		
**Full employed**	*617*	55.1
**In-part employed**	*56*	5.0
**Entrepreneur**	*108*	9.6
**Pension**	*228*	20.3
**Housewife**	*67*	6.0
**Other**	*45*	4.0
**Monthly net income of household per 1 person related to the average (EUR 350)**		
**Significantly lower than average**	*75*	6.7
**Slightly lower than average**	*134*	12.0
**Equal the average**	*312*	27.9
**Slightly above the average**	*366*	32.6
**Significantly higher than average**	*183*	16.4
**Difficult to say**	*50*	4.5
**Class of the locality**		
**Village**	*408*	40.8
**Town up to 20,000 inhabitants**	*131*	13.1
**Town 20,000–50,000 inhabitants**	*107*	10.7
**Town 50,000–100,000 inhabitants**	*81*	8.1
**Town 100,000–200,000 inhabitants**	*77*	7.7
**Town 200,000–500,000 inhabitants**	*90*	9.0
**Town above 500,000 inhabitants**	*106*	10.6

Source: Researchers’ own study

The procedure of the data collection was structured as follows. The invitation to the survey was sent to all members of the GfK research panel who met the recruitment criteria (25,000 persons married or in partnership). The completed questionnaires were collected until the assumed quotas in terms of gender, age, size of the locality, and region of residence were filled. The quotas based on the results of a previously conducted countrywide survey on the randomly selected sample of 1,000 Poles aged 18 years and more (face-to-face interviews at respondents’ home).

The following table presents the sociodemographic structure of the sample.

### Measurement instruments

The factor analysis based on thirteen statements describing different behaviours and principles relating to value orientations indicated four coherent components (Table 2).

**Table 2:** Factor analysis: Rotated component matrix.

	Component 1	Component 2	Component 3	Component 4
**Regular coming to sick unrelated persons’ aid**	0.491			0.469
**Selfless neighbourly help**	0.800			
**Coming to disabled or older persons’ aid on the streets or in buildings**	0.686			
**Coming to friends’ aid in case of e.g. redecoration or removal**	0.760			
**Coming to unknown persons’ aid in case of e.g. showing the way**	0.730			
**Donation for people in need transferred to a foundation/ organization**				0.736
**Food for people in need given directly to the people or through the agency of an official event**				0.608
**I get involved in coming to other persons’ aid if I can have benefits connected with it**			0.733	
**I find that we should not help somebody who is not capable of repaying for the help**			0.742	
**It is not worth devoting time, e.g. a hobby if it is not connected with financial benefits**			0.696	
**Giving makes me happier than receiving**		0.504		
**I have my principles and I never change them**		0.685		
**It happens I help my friends independently of whether I can have benefits from it**		0.585		

Extraction Method: Principal Component Analysis; Rotation Method: Varimax with Kaiser Normalization

Source: Researchers’ own study

Three components might be described as:

- prosocial value orientation characterised by more or less frequent activities: 1) regular coming to sick unrelated persons’ aid; 2) selfless neighbourly help; 3) coming to disabled or older persons’ aid on the streets or in buildings; 4) coming to friends’ aid in case of e.g. redecoration or removal; 5) coming to unknown persons’ aid in case of e.g. showing the way
- reciprocal value orientation characterised by the agreement with the following statements: 1) I get involved in coming to other persons’ aid if I can have benefits connected with it; 2) I find that we should not help somebody who is not capable of repaying for the help; 3) It is not worth devoting time, e.g. a hobby if it is not connected with financial benefits
- egoistic value orientation characterised by the disagreement with the following statements: 1) Giving makes me happier than receiving; 2) It happens that I help my friends independently of whether I can have benefits from it; 3) I have my principles and I never change them.

The scales exhibit a sufficient extent of the convergent validity. The particular items included in the scales show a high level of the mutual correlation (Table 3).

**Table 3:** Correlations between the items included in the scale (N = 1,121).

**Prosocial Value Orientation**				
	**B.**	**C.**	**D.**	**E.**
**A. Regular coming to sick unrelated persons’ aid**	0.356 *	0.409 *	0.305 *	0.314 *
**B. Selfless neighbourly help**		0.482 *	0.547 *	0.506 *
**C. Coming to disabled or older persons’ aid on the streets or in buildings**			0.429 *	0.492 *
**D. Coming to friends’ aid in case of e.g. redecoration or removal**				0.408 *
**E. Coming to unknown persons’ aid in case of e.g. showing the way**				
**Reciprocal Value Orientation**				
	**B.**	**C.**		
**A. I get involved in coming to other persons’ aid if I can have benefits connected with it**	0.402 *	0. 392 *		
**B. I find that we should not help somebody who is not capable of repaying for the help**		0.415 *		
**C. It is not worth devoting time, e.g. a hobby if it is not connected with financial benefits**				
**Egoistic Value Orientation**				
	**B.**	**C.**		
**A. Giving makes me happier than receiving**	0.472 *	0.266 *		
**B. It happens that I help my friends independently of whether I can have benefits from it**		0.277 *		
**C. I have my principles and I never change them**				

*p < 0.001

Source: Researchers’ own study

The scales of prosocial, reciprocal, and egoistic value orientation based on the above factors achieve a satisfying level of reliability (Cronbach’s Alpha: prosocial value orientation = 0.781; reciprocal value orientation = 0.668; egoistic value orientation = 0.600).

The discriminant validity of the scales was examined based on six addition statements which are connected with the construct of the scales in terms of content. Because the statements were measured on the nominal level, the t-student test for two independent samples was used to check if it is possible to reject H0 hypothesis according to which the mean values on the prosocial, reciprocal, and egoistic scales for the subgroups are equal. The results of the analysis are presented in the following table (Table 4).

**Table 4:** Analysis of the discriminant validity of the value orientation scales based on the results of the t-student test.

	Mean Values	Levene’s test	t-Student test
**Statement A–indicator of the prosocial orientation: “It is good to help others, even when you do not get any benefits from it” vs Scale of the Prosocial Value Orientation**	Non-prosocial oriented = 15.3	F = 1.374; p = 0.241	t(1,119) = -3.755; p<0.001
	Prosocial oriented = 16.1		
**Statement B–indicator of the prosocial orientation: “When helping others, you should not even consider whether someone will pay you back or not” vs Scale of the Prosocial Value Orientation**	Non-prosocial oriented = 15.1	F = 6.175; p = 0.013	t(686.579) = -4.673; p<0.001
	Prosocial oriented = 16.2		
**Statement C–indicator of the reciprocal orientation: “It is good to help others, because then you can count on their help” vs Scale of the Reciprocal Value Orientation**	Non-reciprocal oriented = 5.7	F = 1.835; p = 0.176	t(1,119) = -8.480; p<0.001
	Reciprocal oriented = 7.2		
**Statement D–indicator of the reciprocal orientation: “One should not help those who never try to pay back” vs Scale of the Reciprocal Value Orientation**	Non-reciprocal oriented = 6.0	F = 8.589; p = 0.003	t(61.298) = -9.243; p<0.001
	Reciprocal oriented = 8.4		
**Statement E–indicator of the egoistic orientation: “It is good when everyone counts only themselves, because there is no necessity to help them” vs Scale of the Egoistic Value Orientation**	Non-egoistic oriented = 5.4	F = 9.875; p = 0.002	t(55.311) = -6.913; p<0.001
	Egoistic oriented = 7.8		
**Statement F–indicator of the egoistic orientation: “You should only take care of yourself, because you cannot really count on others” vs Scale of the Egoistic Value Orientation**	Non-egoistic oriented = 5.4	F = 32.778; p = 0.001	t(77.536) = -8.316; p<0.001
	Egoistic oriented = 8.0		

Source: Researchers’ own study

The results of the t-student tests makes it possible to reject H0 hypotheses according to which: 1) the mean values on the scale of the prosocial value orientation for persons not oriented prosocially and persons oriented prosocially are equal; 2) the mean values on the scale of the reciprocal value orientation for persons not oriented reciprocally and persons oriented reciprocally are equal; 3) the mean values on the scale of the egoistic value orientation for persons not oriented egoistically and persons oriented egoistically are equal. Hence, the discriminant validity of the built scales can be assumed.

On the next step, because of different length, the variables were submitted to the procedure of recoding to 10 point scales. Finally, the standardised scales of value orientations underwent the stepwise regression analysis in the model taking into consideration dependent variables of compulsive and demonstrative buying and independent variables such as materialism, self-esteem, age, and gender.

The tendency to compensative and compulsive buying was measured by the German Compulsive Buying Indicator consisting of 16 statements assessed on the four-point scale from 1 (“I don’t agree”) to 4 (“I totally agree”); hence, the GCBI amounts to the values between 16 and 64. Following Faber and O’Guinn [26, 30], respondents were qualified as compulsive buyers if they achieved the results on the GCBI scale at least equal to two standard deviations above the mean. Those who achieved the result between one and two standard deviations above the mean value were classified as compensative buyers. Respondents with the result below one standard deviation above the mean were defined as rational consumers.

First, the one-dimensionality, reliability and normal distribution of the results on the GCBI scale were checked. The scale appeared as one-dimensional (KMO above 0.5; Significance of Barlett’s Test of Sphericity p < .05; Eigenvalue of the first Component = 8.23; % of Variance = 51.44). In the factor analysis only one component was extracted and the solution could not be rotated. At the same time, the GCBI scale shows a high degree of internal consistency achieving a very satisfying degree of reliability (Cronbach’s Alpha = 0.94). The coefficients of skewness and kurtosis do not exceed the interval between -1 and +1, thus indicating that the distribution of the GCBI scale is approximately normal (Skewness = 0.30; Kurtosis = -0.55).

The demonstrative type of consumer behaviour was measured on Lange’s scale [9] consisting of five items describing consumers’ attitudes towards brands, novelties on the market or purchase of things which could draw other people’s attention. Respondents assessed on the 5-point Likert’s scale to what extent the statements fitted them personally. Like in case of GCBI, Lange’s scale appeared as one-dimensional (KMO above 0.5; Significance of Barlett’s Test of Sphericity p < 0.05; Eigenvalue of the first Component = 2.64; % of Variance = 52.88) and reliable on the satisfying level (Cronbach’s Alpha = 0.78). Hence, the particular items of Lange’s scale were aggregated at the next step. The coefficients of skewness and kurtosis indicate that the distribution of the aggregated scale is approximately normal (Skewness = -0.157; Kurtosis = -0.80).

The statistical traits of the above scales allow the application of the t-student test for two independent samples, ANOVA procedure, as well as the linear stepwise regression analysis. To sum up, the GCBI as well as Lange’s scale seem to be especially useful to measure compensative, compulsive, and demonstrative buying.

## Results

### Prevalence of compulsive, compensative, and demonstrative buying

The mean value on the GCBI scale achieved 31.08 and the standard deviation 9.26. For this reason, those respondents were qualified as compulsive buyers who achieved the result on the GCBI scale at least on the level of 50. In total, 3.9% of such cases were found in the general population. Those respondents who achieved the result of 41–49 were qualified as compensative buyers. 14.8% of such cases in the whole sample were indicated. In general, 18.7% of adult Poles married or in an informal partnership displayed susceptibility to compensative or compulsive buying.

The demonstrative type of consumer behaviour is significantly more widely prevalent in the Polish society than compensative and compulsive buying. The results on Lange’s scale aggregated to the 5-points Likert’s scale prove that 5.8% of Poles show a very strong susceptibility to demonstrative buying. In total, 1/3 of Poles buy demonstratively more or less often. According to the expectations, demonstrative buying positively correlates with compensative/ compulsive buying. The coefficient of Pearson’s r = 0.327 (p < 0.001) confirms that the stronger susceptibility to demonstrative buying, the greater probability of compensative/ compulsive buying.

### Prevalence of value orientations

The results of the cluster analysis taking into consideration the above described standardised scales show that the biggest segment includes persons representing prosocials. The declarations of almost 2/5 of respondents (38.7%) indicated their belonging to the segment of prosocially oriented persons. About 1/5 of Poles represent the segment of egoists (17.3%). The size of the segment embracing reciprocals is very similar to the last one (16.0%). The remaining part of Poles (28%) represent other types of value orientation.

### Co-dependence of value orientations and consumer behaviours

The results of the t-student test for two independent variables confirm the assumptions that in some cases the examined types of consumer behaviours coexist with the value orientations. The average value on the GCBI scale equals 30.3 among prosocials, while representatives of other value orientations amount to 32.7. The differences between the mean values are statistically significant (t(1,121) = 3.973, p < 0.001). The result allows to point out that susceptibility to compulsive buying is weaker among prosocials than among the persons inclined to other value orientations. In case of demonstrative buying, the statistically significant differences on Lange’s scale between prosocials and members of other segments do not exist.

The reciprocal value orientation seems to coexist with compulsive and demonstrative buying. The mean value of 36.3 is pointed out for reciprocals, whereas persons inclined to other value orientations obtain the mean of 30.9. A similar relation is observable in case of demonstrative buying. Reciprocals achieve the mean on Lange’s scale equalling 16.2, while the interviewed persons not belonging to the segment are characterised by the mean of 14.5. The above presented differences of the mean value are statistically significant (GCBI scale: t(1,121) = -6.602, p < .05; Lange’s scale: t(1,121) = -5.320, p < 0.001).

The egoistic value orientation differentiates susceptibility to compulsive buying, too. While egoists achieve the mean of 34.9, the persons belonging to other segments obtain the result of 31.1 on average. Similarly to the above presented results, the difference is statistically significant (t(1,121) = -5.061; p < 0.001). In case of egoists, the fact of belonging to the segment does not differentiate susceptibility to demonstrative buying statistically significantly.

Because the compensative buying scale does not exist independently of the GCBI scale, the relations between value orientations and susceptibility to compensative buying were examined on the basis of crosstabs. It turns out that belonging to the segment of compensative buyers (15.8%) is differentiated by the reciprocal and the egoistic value orientations, although to a limited extend. Almost 1/4 of reciprocals represent the segment of compensative buyers at the same time (23.8%), whereas the share of representatives of other value orientations is clearly lower (14.3%). The difference is statistically significant, which is evidenced by the Chi-Square Test with the Continuity Correction (Chi2 = 9.603, p < 0.001; Phi = 0.096, p < 0.001). Taking into consideration the 10-point scale of the reciprocal value orientation, the differentiation is still stronger (Chi2 = 139.644; p < 0.001; Cramer’s V = 0.353, p < 0.001). Similar tendencies are observable in case of the egoistic value orientation. More than 1/5 of egoists belong to the segment of compensative buyers (22.2%), while the share of these buyers among representatives of other value orientations amounts to 14.5%. Again, the difference is statistically significant, which is proved by the Chi-Square Test (Chi2 = 6.417, p < 0.001; Phi = 0.079, p < 0.001). Hence, the conclusion can be drawn that representativeness of the reciprocal or the egoistic value orientations coexists with susceptibility to compensative buying to a greater or lesser extent.

Finally, the relations between compulsive as well as demonstrative buying and the value orientations were examined based on the linear stepwise regression analysis (Table 5). The predictors were entered in five steps starting with the variable which explains compulsive and demonstrative buying to the greatest extent. Materialism measured on the Richins and Dawson scale [37] in the Polish version [38] was entered first (Step 1), then self-esteem measured on Rosenberg’s scale [39] (Step 2), followed by age (Step 3), gender (Step 4), and value orientation (Step 5). Because the results referring to the first four steps of the regression analysis were already published elsewhere [12], we are presenting only the data describing steps 4 and 5.

**Table 5:** Summary of the stepwise regression analysis of value orientations as predictors of compulsive buying.

	B	Std. Error	ß	T	*p*
**Step 4**					
**Constant**	28.037	2.666		10.518	0.000
**Materialism**	0.255	0.017	.389	14.856	0.000
**Self-esteem**	-0.537	0.050	-.280	-10.807	0.000
**Age**	-0.043	0.019	-.059	-2.201	0.028
**Gender**	2.294	0.514	.118	4.462	0.000
**Step 5 (1)**					
**Constant**	27.235	2.688		10.134	0.000
**Materialism**	0.254	0.017	0.387	14.828	0.000
**Self-esteem**	-0.552	0.050	-0.288	-11.017	0.000
**Age**	-0.046	0.019	-0.063	-2.346	0.018
**Gender**	2.192	0.515	0.112	4.252	0.000
**Prosocial value orientation**	0.283	0.132	0.054	2.149	0.032
**Step 5 (2)**					
**Constant**	25.656	2.698		9.508	0.000
**Materialism**	0.231	0.018	0.353	12.964	0.000
**Self-esteem**	-0.494	0.050	-0.257	-9.823	0.000
**Age**	-0.033	0.019	-0.046	-1.725	0.085
**Gender**	2.442	0.511	0.125	4.779	0.000
**Reciprocal value orientation**	0.751	0.170	0.119	4.413	0.000
**Step 5 (3)**					
**Constant**	26.585	2.744		9.688	0.000
**Materialism**	0.249	0.017	0.380	14.348	0.000
**Self-esteem**	-0.513	0.051	-0.267	-10.077	0.000
**Age**	-0.040	0.019	-0.055	-2.069	0.039
**Gender**	2.432	0.517	0.125	4.704	0.000
**Egoistic value orientation**	0.307	0.141	0.057	2.171	0.030

Step 4: ΔR^2^ = .324, p < 0.001; Std. Error = 8.020; Step 5 (1): ΔR^2^ = .329, p < 0.001; Std. Error = 8.007; Step 5 (2): ΔR^2^ = .338, p < 0.001; Std. Error = 7.954; Step 5 (3): ΔR^2^ = .329, p < 0.001; Std. Error = 8.006

Source: Researchers’ own study

The results show that materialism and self-esteem are the key variables which explain the appearance of compulsive buying to the greatest extent. If the prosocial value orientation is included in the model, the effect of materialism and self-esteem on the GCBI scale is the same as at step 4. A growth of susceptibility to compulsive buying by 0.254 (+/- 0.017) on the GCBI scale can be assumed along with an increase of materialism by 1 point, while an increase of self-esteem by 1 point means a loss of 0.552 points on the GCBI scale (+/- 0.050). The effect of age and gender on the GCBI scale is weak, but statistically significant. If age increases by 1 year, the result on the GCBI scale decreases by 0.046 (+/- 0.019), while if a person is a woman the result on the GCBI scale should increase by 2.192 (+/- 0.515). Finally, comparing the t-coefficient, the effect of the prosocial value orientation on the GCBI scale seems to be comparable with the effect of age. A growth of susceptibility to compulsive buying by 0.283 (+/- 0.132) on the GCBI scale can be assumed along with an increase of importance of the prosocial value orientation by 1 point. Although the bivariate analysis of the GCBI scale and the prosocial value orientation confirms the previous assumptions, the regression analysis shows some limitations. Indeed, susceptibility to compulsive buying is weaker in prosocials than in the persons preferring other value orientations. But this effect disappears if prosocials are strongly materialistically oriented and suffer from low self-esteem. In general, even though younger women with negative self-esteem who prefer materialism represent the prosocial value orientation, they will be characterised by a stronger susceptibility to compulsive buying than men representing the opposite features.

If the reciprocal value orientation is included in the model, the effect of materialism and self-esteem on the GCBI scale is lesser but still dominant. A growth of susceptibility to compulsive buying by 0.231 (+/- 0.018) on the GCBI scale can be assumed along with an increase of materialism by 1 point, while an increase of self-esteem by 1 point means a loss of 0.494 points on the GCBI scale (+/- 0.050). When the reciprocal value orientation is added, the effect of age becomes statistically insignificant while if the person is a woman the result on the GCBI scale should increase by 2.442 (+/- 0.511). Comparing t-coefficients, the effect of gender on the GCBI scale seems to be comparable with the effect of the reciprocal value orientation. In general, the growth of susceptibility to compulsive buying by 0.751 (+/- 0.170) on the GCBI scale can be assumed along with an increase of importance of the reciprocal value orientation by 1 point. The direction of the observed correlation is coherent with the previously obtained bivariate correlation. Taking into consideration all significant variables, it might be concluded that female reciprocals with negative self-esteem and a preference for materialism at the same time are characterised by a stronger susceptibility to compulsive buying than male reciprocals with healthy self-esteem who reject materialism.

The role of the egoistic value orientation in the explanation of compulsive buying is limited, although statistically significant. If the importance of the egoistic value orientation grows by 1 point, an increase of susceptibility to compulsive buying by 0.307 (+/- 0.141) can be expected. If the egoistic value orientation is included into the model, it does not cause changes of the effect of the remaining variables on the GCBI scale. Still, compulsive buying is explained by materialism and self-esteem mainly. The effect of age in this area is very weak, but statistically significant. Again, the direction of the observed correlation is coherent with the previously obtained bivariate correlation. In general, younger female egoists with negative self-esteem who prefer materialism are characterised by a stronger susceptibility to compulsive buying than older men representing the same value orientation, but characterised by healthy self-esteem and a lesser importance attached to materialism.

In case of demonstrative buying, the primary role of materialism in the regression models is maintained (Table 6). Independently of the step, the growth of susceptibility to demonstrative buying by about 0.100 (+/- 0.008) on Lange’s scale is expected along with each instance of increase of materialism by 1 point. The role of self-esteem in the explanation of demonstrative buying is rather marginal in comparison with the models including compulsive buying. Otherwise than in case of compulsive buying, positive self-esteem is accompanied by demonstrative buying, although this effect is really weak–each case of increase of self-esteem by 1 point causes the growth on the demonstrative buying scale by about 0.05 (+/- 0.023) at steps 2–4. Age does not play any role in the explanation of demonstrative buying. On the contrary, gender seems to be a relatively important factor of demonstrative buying. If the person is a woman, the result on Lange’s scale should decrease by 0.863 (+/- 0.241).

**Table 6:** Summary of the stepwise regression analysis of materialism, self-esteem, age, gender, and value orientations as predictors of demonstrative buying.

	B	Std. Error	ß	t	*p*
**Step 1**					
**Constant**	7.747	0.558		13.883	0.000
**Materialism**	0.097	0.008	0.358	12.831	0.000
**Step 2**					
**Constant**	5.905	1.041		5.673	0.000
**Materialism**	0.103	0.008	0.377	12.856	0.000
**Self-esteem**	0.049	0.023	0.061	2.095	0.036
**Step 3**					
**Constant**	5.964	1.143		5.217	0.000
**Materialism**	0.103	0.008	0.377	12.706	0.000
**Self-esteem**	0.049	0.023	0.062	2.095	0.036
**Age**	-0.001	0.009	-0.004	-0.124	0.901
**Step 4**					
**Constant**	7.817	1.249		6.257	0.000
**Materialism**	0.101	0.008	0.372	12.591	0.000
**Self-esteem**	0.052	0.023	0.065	2.237	0.025
**Age**	-0.013	0.009	-0.043	-1.412	0.158
**Gender**	-0.863	0.241	-0.107	-3.584	0.000
**Step 5 (1)**					
**Constant**	7.095	1.252		5.665	0.000
**Materialism**	0.100	0.008	0.369	12.586	0.000
**Self-esteem**	0.039	0.023	0.049	1.660	0.097
**Age**	-0.016	0.009	-0.052	-1.736	0.083
**Gender**	-0.955	0.240	-0.118	-3.977	0.000
**Prosocial value orientation**	0.254	0.061	0.116	4.146	0.000
**Step 5 (2)**					
**Constant**	7.201	1.272		5.660	0.000
**Materialism**	0.095	0.008	0.350	11.310	0.000
**Self-esteem**	0.063	0.024	0.080	2.672	0.008
**Age**	-0.010	0.009	-0.035	-1.143	0.253
**Gender**	-0.825	0.241	-0.102	-3.425	0.001
**Reciprocal value orientation**	0.194	0.080	0.074	2.420	0.016
**Step 5 (3)**					
**Constant**	8.747	1.284		6.814	0.000
**Materialism**	0.105	0.008	0.386	12.946	0.000
**Self-esteem**	0.037	0.024	0.046	1.533	0.126
**Age**	-0.014	0.009	-0.048	-1.594	0.111
**Gender**	-0.952	0.242	-0.118	-3.936	0.000
**Egoistic value orientation**	-0.196	0.066	-0.088	-2.972	0.003

Step 1: R^2^ = .128, p < 0.001; Std. Error = 3.78215; Step 2: ΔR^2^ = .132, p < 0.001; Std. Error = 3.776; Step 3: ΔR^2^ = .132, p < 0.001; Std. Error = 3.778; Step 4: ΔR^2^ = .142, p < 0.001; Std. Error = 3.758; Step 5 (1): ΔR^2^ = .155, p < 0.001; Std. Error = 3.731; Step 5 (2): ΔR^2^ = .146, p < 0.001; Std. Error = 3.750; Step 5 (3): ΔR^2^ = .148, p < 0.001; Std. Error = 3.745

Source: Researchers’ own study

The role of value orientations in the explanation of demonstrative buying is relatively limited. The prosocial value orientation seems to be the strongest predictor of demonstrative buying among all the analysed value orientations. The growth of susceptibility to demonstrative buying by 0.254 (+/- 0.061) on Lange’s scale can be assumed together with an increase of importance of the prosocial value orientation by 1 point. Self-esteem and age do not play any significant role in this model. Interesting conclusions can be drawn comparing the results with the previously presented bivariate analysis. According to the latter one, hypothesis “zero” assuming no statistically significant differences between prosocials and representatives of other value orientations has been confirmed. The regression analysis finds out that the correlation appears if a prosocially oriented person is a man preferring materialism.

The direction of the effect triggered by the reciprocal value orientation is similar, although the power of the effect is weaker. Susceptibility to demonstrative buying is growing by 0.194 (+/- 0.080) on Lange’s scale along with an increase of importance of the reciprocal value orientation by 1 point. Again, age does not play any significant role in the model. In contrast, self-esteem seems to intensify susceptibility to demonstrative buying to a similar extent as the reciprocal value orientation. In this case, the direction of the observed correlation is coherent with the previously obtained bivariate analysis. In general, male reciprocals with positive self-esteem and preferring materialism to a greater or lesser extent are characterised by a greater susceptibility to demonstrative buying than women characterised by the opposite features.

The effect of the egoistic value orientation on demonstrative buying is quite opposite in comparison with other value orientations. The previously conducted bivariate analysis does not evidence any statistically significant differences between susceptibility to demonstrative buying of people oriented egoistically and preferring other value orientations. The multidimensional regression analysis shows quite a different picture. A correlation between the egoistic value orientation and demonstrative buying appears if materialism is preferred by men. Then, the probability of demonstrative buying becomes weaker by 0.196 (+/- 0.066) if the importance of the egoistic value orientation grows by 1 point. Similarly to the model including the prosocial value orientation, self-esteem and age do not play any role in the explanation of susceptibility to demonstrative buying.

## Discussion

In accordance with our expectations, we observe the phenomenon that some value orientations coexist with some chosen types of consumer behaviour. Susceptibility to compensative buying is higher among reciprocals and egoists than among persons who prefer other value orientations. According to the results of the cluster analysis, 1/3 of Poles married or in a partnership are characterised by the co-dependence of reciprocal or egoistic value orientations and compensative buying. The same conclusion can be drawn about compulsive buying—susceptibility to this type of consumer behaviour seems to be higher among reciprocals and egoists than among persons choosing other value orientations. In contrast, the prosocial value orientation appears to be an obstacle of compulsive buying: Prosocials’ susceptibility to compulsive buying is weaker compared with persons representing other value orientations. Finally, susceptibility to demonstrative buying is observable among reciprocals to a greater extent than among representatives of other value orientations. It means that the co-dependence of the reciprocal value orientation and demonstrative buying might appear among about 1/6 of Poles married or in a partnership.

Reasons for the co-dependence of the particular value orientations and consumer behaviours can be looked for in specific social and cultural conditions.

Firstly, relatively strong bonds between value orientations and religiosity in the Polish society might be expected. From the sociological point of view, this connection between value orientations and religiosity is quite obvious. According to Malinowski [40], each religion, primitive or not, consists of three dimensions–dogmatic, ritual, and ethical. The praxis of religious rituals requires a community, which leads to an establishment of social cohesion because the praxis has to be based on interpersonal trust, community spirit, and common actions. Contemporary conclusions about the relations between religiosity and value orientations stay in line with Malinowski’s observation of primitive beliefs and conduct. Schwartz and Huismans [41] proposed a hypothetical model of correlations between religiosity and the importance of values assuming that religious people prefer the values of universalism, benevolence, and tradition compared with non-religious persons who tend to underline the importance of hedonism and achievement more often than the first group. When referring to the above concept, Schnabel and Groetsch [42] analysed the data concerning the dimensions of religiosity and value orientation across the European countries. They pointed out relatively stable pattern independent of a nationality: Religious people are more conservative and appreciative of social norms in their lives than non-religious persons. “Our results, however, suggest that religion and individual religiousness support in particular commitment to tradition and conformity” [42, pp. 175, 176].

This statement by Schnabel and Groetsch is of high importance for the presented study. Compulsive, compensative, as well as demonstrative consumer behaviours might be perceived as signs of consumerism from the Christian point of view. In principle, consumerism as an idea and its different empirical symptoms such as excessive buying are disapproved by Christianity. On the other hand, the type of the prosocial value orientation corresponds with the Christian ethics appealing to the principle of the love of neighbour. Hence, susceptibility to compensative, compulsive or demonstrative buying should be lower in those societies where Christianity plays an important role. For the same reasons, prosocials in these societies should reject compensative, compulsive, and demonstrative buying to a greater extent than supporters of other types of value orientations. According to the results of the World Values Survey, the rate of Poles who define themselves as religious amounts to the level of 83%. The ratio is about 1.5–2.0 times higher compared e.g. with the USA (58%), Germany (52%), Spain (47%), or the Netherlands (42%). In total, only 10% of Poles declare that they never attend religious services. The ratio in the compared countries is even a few times higher: the Netherlands (57%), Spain (46%), Germany (41%), the USA (33%) [43]. The relatively large prevalence of religious practices among Poles may be one factor strengthening the prevalence of the prosocial value orientation, which holds back the development of consumerism and compensative, compulsive as well as demonstrative buying in this connection. The obtained data confirm this assumption. The results of the t-test for two independent samples show that susceptibility to compulsive buying among prosocials is lower than among representatives of other types of value orientation. From this point of view, the prosocial value orientation “protects” against compulsive buying to a greater or lesser extent. This protection becomes weaker if the main factors of compulsive buying begin to play an important role. The prosocial value orientation does not protect an individual against compulsive buying if they are strongly materialistically oriented and characterised by lower self-esteem at the same time. A similar development is observable in case of demonstrative buying. Although no statistically significant bivariate correlations exist between the prosocial value orientation and demonstrative buying, the co-dependence of both variables appears among men preferring materialism.

Secondly, in a sense, the correlation of value orientations and consumer behaviours was inherited from Poland’s socialist period in the 1980s characterised by a drastically insufficient degree of satisfying the citizens’ economic needs [44]. This fact, connected with a very high acclaim of the western pop culture as a sign of freedom led to the prevalent acceptance of brands as a means of social differentiation and a source of social prestige. It does not surprise that demonstrative buying develops first of all among representatives of value orientations characterised by strong concentration on keeping the social exchange in balance and by the use of consumer goods to manifest one’s own identity or to manifest one’s own social position. As a consequence, consumerism comes upon a breeding ground in these segments. This leads to the development of susceptibility to compensative buying as a reaction against inevitable failures of the strategy using the symbolic values of consumer goods to reach meaning and satisfaction in life. Finally, excessive compensative buying might lead to the development of compulsive buying.

Thirdly, the causes of the co-dependence between particular value orientations and consumer behaviours can be found in specific upbringing styles. Specific conditions of socialisation and authoritarian or overprotective upbringing styles which lead to a distortion of an individual’s autonomy and a healthy feeling of one’s own value are mentioned as the main social reasons for susceptibility to compensative/ compulsive buying [45]. Reasons for the development of the egoistic value orientation might be looked for in the same conditions. The authoritarian or overprotective upbringing styles depriving an individual autonomy and healthy self-esteem can influence the development of the egoistic value orientation as a specific reaction defending autonomy and improving self-esteem.

Fourthly, the reciprocal value orientation is characteristic of persons oriented more traditionally in reference to the establishment of social relationships. In part, this type of persons belongs to the generations born in the early 1970s at the latest. They had the highest chance to internalize the reciprocal value orientation because their childhood and adolescence fell on the period before the intensive modernization processes after 1989. The generations suffered from insufficient satisfaction of their economic needs at the same time, which might lead to a specific personal value hierarchy in which materialistic values are more or less on the top [46]. Actually, the generations which internalized the reciprocal value orientation might practise consumer behaviours like excessive buying (connected with demonstrative, compensative, and compulsive buying), which allows satisfying their economic needs to a subjectively sufficient extent.

## Conclusions

The conducted study points to the possibility of relations existing between different types of value orientations and consumer behaviours. The obtained data confirm hypotheses H2, H3, and H4. Indeed, the stronger the reciprocal value orientation, the stronger susceptibility to compensative, compulsive as well as demonstrative buying. The stronger the egoistic orientation, the stronger susceptibility to compensative and compulsive buying. In contrast, hypothesis H1 has to be rejected; the obtained data do not indicate that the prosocial value orientation differentiates susceptibility to compensative buying.

In part, the characteristic of the above presented correlations are modified in the multidimensional models. Still, materialism and self-esteem are the main factors of compulsive buying. If both variables are included in the model, the negative correlation between the prosocial value orientation and compulsive buying disappears. Furthermore, even prosocials tend towards compulsive buying if they suffer from lower self-esteem and prefer materialism. Similar conclusions can be drawn in case of the egoistic value orientation and demonstrative buying. There does not exist any bivariate correlation between both variables. If materialism is included in the model, the egoistic orientation seems to weaken susceptibility to demonstrative buying but only among men.

It is worth putting the question about the future development of the correlations between the analysed types of value orientations and consumer behaviours. In total, four basic directions of the changes are characteristic of the Polish society: 1) secularization understood as an autonomization of two areas of social life–value orientations and religiosity; 2) an individual’s growing autonomy from the pressure of tradition, customs, and external authorities; 3) social change of value orientations; 4) desecularization (revival of religiosity) or reconstruction of moral bonds within the development of civic society [47]. It remains an open question if the processes of secularization and individualization strengthen the prevalence of the reciprocal and the egoistic value orientations instead of the prosocial value orientation, which finally would lead to the development of consumerism and its signs–compensative, compulsive, and demonstrative buying.

Some limitations should be kept in mind when analysing the results of the study. Firstly, the sample of the survey embraces only the respondents who are married or in an informal partnership, which raises the question about statistical representativeness of the results for the whole society. The 2016 study conducted on the statistically representative sample of Poles aged 15 and over pointed out a 4.4% rate of compulsive buyers [48]. Because the difference compared with the current study equals 1.5% and does not exceed the statistical error, the sample seems to be more or less representative for the whole society.

Secondly, the data could be influenced by the phenomenon of social desirability bias, first of all in case of the older respondents. This effect is observable in the social research, especially if the topic of addiction is interviewed. The presented conclusions about compulsive buying base on the declarations, not on the actual behaviour, which causes a growing danger of the social desirability bias effect. This effect is probably greater in case of the older than the younger generation because the latter one is characterised by a weaker susceptibility to yielding to social control.

Thirdly, the study is not longitudinal, hence, the conclusions consider the co-existence of the independent and dependent variables but not any causal relations.

## Supporting information

S1 Questionnaire Questionnaire ENG. (DOCX)

S2 Questionnaire Questionnaire PL. (DOCX)
