# Supplementary material for: Links between types of value orientations and consumer behaviours. An empirical study
Source: PLoS One. 2022 Feb 24;17(2):e0264185. doi: 10.1371/journal.pone.0264185 (PMC8870570; doi:10.1371/journal.pone.0264185)
Supplement: S1 Questionnaire — (DOCX) [file pone.0264185.s001.docx]

**245 627**

**2017-06-22**

*Length of interview: 20 minutes*

*Start fieldwork: 28 June, 2017*

*End fieldwork: 3 July, 2017*

**I. SAMPLE VARIABLES**

*N/A*

**II. QUOTA CHECK BASED ON SAMPLE VARIABLES**

*N/A*

**III. INTRODUCTION**

**SCREENER**

S1 [S] **Please tick your gender:**

1. Man

2. Woman

S3

**How old are you?**

*SCRIPTER: Show three-digit box to write in the answer.*

*SCRIPTER: Hidden box („No answer”).*

**QUOTA CHECK**

**Main questionnaire**

Base: all respondents

Q1 [S in each row]

**There are listed different help acts below which are conducted by people more or less often. Did you help somebody in this way?**

*SCRIPTER: Rotation of the statements*.

*In rows:*

1. Regular coming to sick unrelated persons’ aid

2. Selfless neighbourly help

3. Coming to disabled or older persons’ aid on the streets or in buildings

4. Coming to friends’ aid in case of e.g. redecoration or removal
5. Coming to unknown persons’ aid in case of e.g. showing the way
6. Donation for people in need transferred to a foundation/ organization
7. Food for people in need given directly to the people of through the agency of an official event

*In columns:*

1. No, never
2. Yes, once or twice
3. Yes, but rather seldom
4. Yes, relatively often
5. Yes, very often

Base: all respondents

Q2. [S]

**Different opinions exist about helping other people. Which of the opinions presented below suit you the best?**

1. It is good when everyone counts only themselves, because there is no necessity to help them

2. It is good to help others, because then you can count on their help

3. It is good to help others, even when you do not get any benefits from it

Base: all respondents

Q3. [S in each row]

**There are a few statements describing different situations. Please tick at each statement to what extent do you agree with it or not.**

*SCRIPTER: Rotation of the statements*.

*In rows:*

1. I get involved in coming to other persons’ aid if I can have benefits connected with it
2. I find that we should not help somebody who is not capable of repaying for the help
3. Giving makes me happier than receiving
4. It is not worth devoting time, e.g. a hobby if it is not connected with financial benefits
6. I have my principles and I never change them
7. It happens I help my friends independently of whether I can have benefits from it

*In columns:*

1. Definitely I disagree with it
2. I rather disagree with it
3. Difficult to say
4. I rather agree with it
5. Definitely I agree with it

Base: all respondents

Q4. [S]

**It happens that we help another person and he/she does not repay, although he/she has an opportunity. Which of these statements do you agree with the most?**

1. One should not help those who never try to pay back

2. When helping others, you should not even consider whether someone will pay you back or not

Base: all respondents

Q5. [S in each pair of statements]

**If you had to choose one of the two statements below, which one would you choose?**

1. It is worth helping others, because you never know when you will need help yourself

2. You should only take care of yourself, because you cannot really count on others

Base: all respondents

Q17. [S in each row]

**To what extent do the statements fit to you or not?**

*SCRIPTER: Rotation of the statements*.

*In rows:*

1. Generally, I pay attention to the fact which firm produces the good purchased by me
2. I buy so-called brand products willingly
3. I struggle to get to know about novelties on the market
4. If I buy something I pay attention whether the items will be assessed positively by my friends
5. I buy the items which can effect on my friends

*In columns:*

1. Definitely it does not fit me
2. It rather does not fit me
3. It is difficult to say
4. It rather fits me
5. Definitely it fits me

Base: all respondents

Q18. [S in each row]

**And now still one list of statements. To what extent does each of statements fit you or not?**

**SCRIPTER ROTATION**

*In rows:*

1. I often have an unexplainable urge, a sudden and spontaneous desire, to go and buy sometimes in a store

2. At times, I have felt somewhat guilty after buying a product

3. There are times when I have a strong urge to buy

4. I sometimes feel that something inside of me pushed me to go shopping

5. Often I buy something just because it is cheap

6. Often I buy something because simply I feel like buying

7. Often I have a feeling that I absolutely must have an item

8. There are some things I buy that I do not show to anybody for fear of being perceived as irrational in my buying behaviour

9. Often I ask myself after a purchase of an item if the purchase was really so important

10. As soon as I walk down streets or I enter a shopping centre, I have an irresistible urge to go into a shop to buy something

11. When I have money, I cannot help but spend part of the whole of it

12. I am rather free-spending

13. I am one of those people who often responds to direct mail offers/ websites of online stores

14. For me, shopping is a way of facing the stress of my daily life and of relaxing

15. I have often bought something which I do not use at all

16. I have often bought a product that I did not need, while knowing I had very little money left

*In columns:*

1. I disagree

2. I rather disagree

3. I rather agree

4. I totally agree

Base: all respondents

Q19. [S in each row]

**Please think for a moment about the things that belong to you and consider what they do in your life, what they mean for you. Then, please mark on the scales to what extent you agree with each of the following statements. There are no right or wrong answers here, we want to identify best your true beliefs and opinions.**

*SCRIPTER: Rotation of the statements*.

*In rows:*

1. I put less emphasis on material things than most people I know
2. Some of the most important achievements in life include acquiring material possessions
3. I have all the things I really need to enjoy life
4. The things I own say a lot about how well I’m doing in life
5. I’d be happier if I could afford to buy more things
6. I like to own things that impress people
7. I don’t pay much attention to the material objects other people own
8. It sometimes bothers me quite a bit that I can’t afford to buy all the things I’d like
9. The things I own allow me to feel well among my friends
10. The possession of some material goods might breathe confidence into myself
11. The material goods do not evidence the life success
12. The possession of material goods might provide sense of freedom and independency
13. I strive persistently to acquire material goods and objects that I dream about
14. I have many such things that could not be replaced by others
15. Having or not having certain goods does not affect my self-esteem
16. I enjoy just having certain things
17. I wouldn’t be any happier if I owned nicer things
18. Collecting goods and money is a pleasure for me
19. I admire people who own expensive homes, cars, and clothes etc.
20. I like a lot of luxury in my life

*In columns:*

1. I definitely disagree
2. I disagree
3. I rather disagree
4. Difficult to say

5. I rather agree

6. I agree

7. I definitely agree

Base: all respondents

Q20. [S in each row]

**Below is a list of statements dealing with your general feelings about yourself. Please indicate how strongly you agree or disagree with each statement.**

*SCRIPTER: Rotation of the statements*.

*In rows:*

1. I feel that I’m a person of worth, at least on an equal plane with others

2. I feel that I have a number of good qualities

3. All in all, I am inclined to feel that I am a failure

4. I am able to do things as well as most other people

5. I feel I do not have much to be proud of

6. I take a positive attitude toward myself

7. On the whole, I am satisfied with myself

8. I wish I could have more respect for myself

9. I certainly feel useless at times

10. At times I think I am no good at all

*In columns:*

1. Strongly disagree
2. Rather disagree
3. Rather agree
4. Strongly agree
