# Supplementary material for: Links between types of value orientations and consumer behaviours. An empirical study
Source: PLoS One. 2022 Feb 24;17(2):e0264185. doi: 10.1371/journal.pone.0264185 (PMC8870570; doi:10.1371/journal.pone.0264185)
Supplement: S2 Questionnaire — (DOCX) [file pone.0264185.s002.docx]

**245 627**

**2017-06-22**

*Length of interview: 20 minutes*

*Start fieldwork: 28 June, 2017*

*End fieldwork: 3 July, 2017*

**I. SAMPLE VARIABLES**

*N/A*

**II. QUOTA CHECK BASED ON SAMPLE VARIABLES**

*N/A*

**III. INTRODUCTION**

**SCREENER**

S1 [S] **Proszę zaznaczyć swoją płeć:**

1. Mężczyzna

2. Kobieta

S3

**Ile ma Pan(i) skończonych lat?**

*SCRIPTER: Show three-digit box to write in the answer.*

*SCRIPTER: Hidden box („Brak odpowiedzi”).*

**QUOTA CHECK**

**Main questionnaire**

Base: all respondents

P1 [S in each row]

**Poniżej przedstawione są różne sytuacje, które zdarzają się ludziom częściej lub rzadziej. Czy zdarzają się Panu(i) takie sytuacje?**

*SCRIPTER: Rotation of the statements*.

*In rows:*

1. Systematyczna pomoc chorej osobie spoza najbliższej rodziny

2. Bezinteresowna pomoc sąsiedzka

3. Pomoc osobom niepełnosprawnym/ starszym na ulicy lub w budynkach, np. przy przejściu przez ulicę, przy wsiadaniu do autobusu, do windy, przy otwieraniu drzwi itp.
4. Pomoc udzielana przyjaciołom/ znajomym, np. przy przeprowadzce, remoncie, naprawach itp.
5. Bezinteresowna pomoc nieznajomej osobie, np. wskazanie drogi, podwiezienie samochodem, udzielenie pierwszej pomocy
6. Datek finansowy dla potrzebujących przekazany jakiejś fundacji/ organizacji
7. Datek żywnościowy dla potrzebujących przekazany prywatnie lub w ramach takich akcji jak Szlachetna Paczka, Pomóż Dzieciom Przetrwać Zimę

*In columns:*

1. Nie, nie zdarzyło się
2. Zdarzyło się, raz czy dwa
3. Zdarzało się, ale rzadko
4. TAK, stosunkowo często
5. TAK, bardzo często

Base: all respondents

P2 [S]

**Na temat udzielania pomocy innym ludziom istnieją różne opinie. Która z przedstawionych poniżej opinii odpowiada Panu(i) najbardziej?**

1. Dobrze jest, gdy każdy liczy tylko na siebie, ponieważ nie ma konieczności pomagania mu

2. Dobrze jest pomagać innym, bo można wtedy liczyć na pomoc z ich strony

3. Dobrze jest pomagać innym, nawet wtedy, gdy samemu nic się z tego nie ma

Base: all respondents

P3. [S in each row]

**Poniżej znajduje się kilka stwierdzeń opisujących różne sytuacje. Przy każdym stwierdzeniu proszę zaznaczyć, w jakim stopniu zgadza lub nie zgadza się Pan(i) z nim. Odpowiedzi proszę udzielić posługując się skalą od 1 do 5, gdzie 1 oznacza, że zdecydowanie NIE zgadza się Pan(i) z tym stwierdzeniem, a 5, że zdecydowanie się Pan(i) z nim zgadza.**

*SCRIPTER: Rotation of the statements*.

*In rows:*

1. Angażuję się w pomoc innym, jeśli coś z tego mogę mieć
2. Uważam, że nie należy pomagać komuś, kto nie potrafi się odwdzięczyć
3. Zgadzam się z zasadą „więcej radości jest w dawaniu, aniżeli w braniu”
4. Nie warto poświęcać czasu np. jakiemuś hobby, jeśli nie przynosi to finansowych korzyści
6. Mam swoje zasady postępowania i nie zmieniam ich pod wpływem znajomych i otoczenia
7. Staram się pomagać znajomym niezależnie od tego, czy mogę coś z tego mieć

*In columns:*

1. Zdecydowanie NIE zgadzam się
2. Raczej NIE zgadzam się
3. Trudno powiedzieć
4. Raczej zgadzam się
5. Zdecydowanie zgadzam się

Base: all respondents

P4 [S]

**Zdarza się, że pomagamy innemu człowiekowi, a ten nie odwdzięcza się, chociaż ma okazję. Z którym z tych stwierdzeń zgadza się Pan(i) najbardziej?**

1. Nie należy pomagać tym, którzy nigdy nie starają się odwdzięczyć

2. Pomagając innym, nie powinno się w ogóle brać pod uwagę, czy ktoś się odwdzięczy, czy nie

Base: all respondents

P5 [S in each pair of statements]

**Gdyby musiał(a) Pan(i) wybrać jedno z dwóch poniższych twierdzeń, to które by Pan(i) wybrał(a)?**

1. Warto pomagać innym, bo nigdy nie wiadomo, kiedy samemu będzie się pomocy potrzebować

2. Należy troszczyć się tylko o siebie, bo tak naprawdę na innych nie można liczyć

Base: all respondents

P17. [S in each row]

**W jakim stopniu poniższe stwierdzenia pasują bądź nie pasują do Pana(i)?**

*SCRIPTER: Rotation of the statements*.

*In rows:*

1. Generalnie zwracam uwagę na to, jaka firma wyprodukowała produkt, który kupuję
2. Chętnie kupuję tzw. produkty markowe
3. Staram się dowiadywać o nowościach na rynku
4. Gdy coś kupuję zwracam uwagę na to, by kupione rzeczy zostały później pozytywnie ocenione przez moich przyjaciół/ znajomych
5. Kupuję takie rzeczy, którymi mogę zrobić wrażenie na przyjaciołach/ znajomych

*In columns:*

1. Zdecydowanie nie pasuje do mnie
2. Raczej nie pasuje do mnie
3. Trudno powiedzieć
4. Raczej pasuje do mnie
5. Zdecydowanie pasuje do mnie

Base: all respondents

P18. [S in each row]

**I jeszcze jedna lista stwierdzeń. W jakim stopniu każde z tych stwierdzeń pasuje bądź nie pasuje do Pana(i)?**

*SCRIPTER: Rotation of the statements*.

*In rows:*

1. Często kupuję coś tylko dlatego, że jest tanie
2. Czasami widzę coś i czuję, że muszę to kupić
3. Zdarzało mi się często kupić coś, czego potem w ogóle nie używałem
4. Często po zakupie jakiejś rzeczy pytam samego siebie, czy rzeczywiście ten zakup był taki ważny
5. Często mam poczucie, że jakąś rzecz muszę bezwzględnie mieć
6. Czasami mam wyrzuty sumienia, gdy coś sobie kupię
7. Często kupuje coś, gdy po prostu mam ochotę na kupowanie
8. Czasami zauważam, że coś mnie popycha do tego, by iść na zakupy
9. Zdarzało mi się często kupić coś, na co właściwie w ogóle nie mogłem sobie pozwolić
10. Czasami bez szczególnej przyczyny nagle czuję, ze muszę wyjść z domu i pójść na zakupy
11. Robię zakupy, by uciec od niemiłej codzienności i się zrelaksować
12. Gdy idę przez miasto albo przez centrum handlowe/ sklep, czuje silną potrzebę, by coś sobie kupić
13. Gdy mam pieniądze, czuję, że muszę je wydać
14. Jestem raczej rozrzutny
15. Katalogi sprzedaży wysyłkowej/strony sklepów internetowych są dla mnie dużą pokusą, po ich obejrzeniu najczęściej wysyłam zamówienie
16. Często nie mam odwagi pokazać kupionych rzeczy innym osobom, gdyż mogliby mnie uznać za osobę nierozsądną

*In columns:*

1. Całkowicie nie pasuje do mnie
2. Raczej nie pasuje do mnie
3. Raczej pasuje do mnie
4. Całkowicie pasuje do mnie

Base: all respondents

P19. [S in each row]

**Proszę pomyśleć przez chwilę o rzeczach, które do Pana(i) należą i zastanowić się, jaką pełnią one funkcję w Pana(i) życiu, jakie mają dla Pana(i) znaczenie. Następnie proszę zaznaczyć na skalach, w jakim stopniu zgadza się Pan(i) z każdym z podanych poniżej stwierdzeń. Nie ma tu odpowiedzi dobrych lub złych, chodzi nam o to, by Pan(i) najlepiej określić swoje prawdziwe przekonania i opinie.**

*SCRIPTER: Rotation of the statements*.

*In rows:*

1. Posiadanie dóbr materialnych ma dla mnie mniejsze znaczenie niż dla większości ludzi, których znam
2. Uważam, że jedno z najważniejszych osiągnięć życiowych polega na zdobyciu wysokiej pozycji materialnej
3. W sensie materialnym mam wszystko, czego potrzebuję, by cieszyć się życiem
4. Zasób posiadanych dóbr nie jest oznaką sukcesu życiowego
5. Sądzę, że byłbym szczęśliwszy, gdybym miał lepsze rzeczy, niż aktualnie posiadam
6. Lubię mieć przedmioty, które robią duże wrażenie na innych
7. Nie zwracam szczególnej uwagi na to, co inni ludzie posiadają
8. Czasem bardzo doskwiera mi to, że nie mogę mieć wszystkich tych rzeczy, które chciałbym posiadać
9. Przedmioty, które mam, pozwalają mi dobrze czuć się wśród moich przyjaciół i znajomych
10. Posiadanie pewnych przedmiotów materialnych może dodawać pewności siebie
11. Zgromadzone dobra materialne nie świadczą o moim sukcesie i powodzeniu życiowym lub ich braku
12. Posiadanie dóbr materialnych może zapewnić poczucie wolności i niezależności
13. Usilnie dążę do zdobycia dóbr i przedmiotów materialnych, o których marzę
14. Mam wiele takich rzeczy, których nie dałoby się zastąpić innymi
15. Fakt posiadania lub nieposiadania pewnych dóbr nie wpływa na moją samoocenę
16. Cieszy mnie już samo posiadanie pewnych rzeczy
17. Nie byłbym bardziej zadowolony, gdybym mógł pozwolić sobie na kupowanie droższych rzeczy
18. Gromadzenie dóbr i pieniędzy jest dla mnie przyjemnością
19. Ludzie, którzy posiadają kosztowne przedmioty, domy, samochody itp., budzą we mnie podziw i respekt.
20. Luksus to jedna z najważniejszych wartości w moim życiu

*In columns:*

1. Zdecydowanie nie zgadzam się
2. Nie zgadzam się
3. Raczej nie zgadzam się
4. Trudno powiedzieć

5. Raczej się zgadzam

6. Zgadzam się

7. Zdecydowanie zgadzam się

Base: all respondents

P20. [S in each row]

**Poniżej znajdują się różne stwierdzenia, które dotyczą Pana(i) przekonań o samym sobie. Proszę wskazać, w jakim stopniu zgadza się bądź nie zgadza Pan(i) z każdym z tych twierdzeń. Proszę postarać się określić to, co naprawdę Pan(i) sądzi na swój temat. Liczą się tylko szczere odpowiedzi.**

*SCRIPTER: Rotation of the statements*.

*In rows:*

1. Uważam, że jestem osobą wartościową przynajmniej w takim samym stopniu, co inni

2. Uważam, że posiadam wiele pozytywnych cech

3. Ogólnie biorąc jestem skłonny(a) sądzić, że nie wiedzie mi się

4. Potrafię robić różne rzeczy tak dobrze, jak większość innych ludzi

5. Uważam, że nie mam wielu powodów, aby być z siebie dumnym(ą)

6. Lubię siebie

7. Ogólnie rzecz biorąc, jestem z siebie zadowolony(a)

8. Chciał(a)bym mieć więcej szacunku dla samego siebie

9. Czasami czuję się bezużyteczny(a)

10. Niekiedy uważam, że jestem do niczego

*In columns:*

1. Zdecydowanie nie zgadzam się
2. Raczej nie zgadzam się
3. Raczej zgadzam się
4. Zdecydowanie zgadzam się
